# Reticulate Evolution and Marine Organisms: The Final Frontier?

**DOI:** 10.3390/ijms10093836

**Published:** 2009-09-03

**Authors:** Michael L. Arnold, Nicole D. Fogarty

**Affiliations:** 1 Department of Genetics, University of Georgia, Athens, GA 30602, USA; 2 Department of Biological Science, Florida State University, Tallahassee, Florida 32306-4295, USA; E-Mail:fogarty@bio.fsu.edu (N.D.F.)

**Keywords:** introgression, horizontal transfer, web of life, marine

## Abstract

The role that reticulate evolution (*i.e.,* via lateral transfer, viral recombination and/or introgressive hybridization) has played in the origin and adaptation of individual taxa and even entire clades continues to be tested for all domains of life. Though falsified for some groups, the hypothesis of divergence in the face of gene flow is becoming accepted as a major facilitator of evolutionary change for many microorganisms, plants and animals. Yet, the effect of reticulate evolutionary change in certain assemblages has been doubted, either due to an actual dearth of genetic exchange among the lineages belonging to these clades or because of a lack of appropriate data to test alternative hypotheses. Marine organisms represent such an assemblage. In the past half-century, some evolutionary biologists interested in the origin and trajectory of marine organisms, particularly animals, have posited that horizontal transfer, introgression and hybrid speciation have been rare. In this review, we provide examples of such genetic exchange that have come to light largely as a result of analyses of molecular markers. Comparisons among these markers and between these loci and morphological characters have provided numerous examples of marine microorganisms, plants and animals that possess the signature of mosaic genomes.

## 1. Introduction

**Figure 1.**
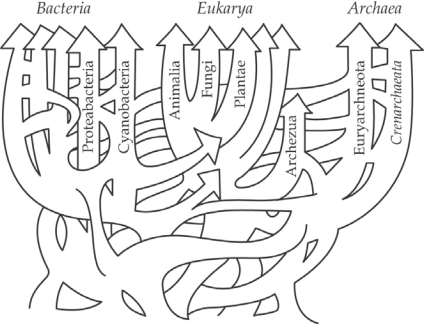
A representation of the history of biological diversification of all life, reflecting the role of introgressive hybridization and lateral exchange in the development of new lineages with mosaic genomes (from [8], as modified in [6]). Reprinted with permission from The American Association for the Advancement of Science [8].

The occurrence of reticulate evolution (*i.e.,* involving the processes of natural hybridization, horizontal transfer and viral recombination) is now well established as having affected the origin and adaptation of organisms from all of the domains of life (see [1–10] for reviews). The evaluation of available data sets, particularly those involving molecular markers, has thus led to the falsification of the hypothesis that most lineages have arisen and evolved in genetic isolation from other lineages (*i.e.,* the allopatric model; see [6,7] for reviews). Instead, models of evolution, that incorporate divergence in the face of gene flow, have been repeatedly supported as more and more genomes have been examined in detail (*e.g.,* [11–13]). Indeed, such is the phylogenetic extent of the genetic exchange that has been detected that we have argued [6,7] for the substitution of the tree-of-life metaphor with one best described as a web-of-life (Figure 1). Such a metaphor thus incorporates introgressive hybridization, lateral exchange and natural selection in the development of evolutionary lineages possessing mosaic genomes (*i.e.,* genomes made up of elements from multiple evolutionary lineages; [6,7]).

In contrast to the above conclusions, some authors have suggested that the extent to which reticulate evolution has affected marine organisms is limited. For example, Arnold [5] agreed with the conclusion of Hubbs [14] that the available data supported “*…the hypothesis that natural hybridization is less common in marine fishes.*” This conclusion seems to be substantiated by the relative uniqueness of findings such as those reported by Roques *et al.* [15] in their paper on introgressive hybridization in redfish (genus *Sebastes*), which they referred to as “*a rare marine example*”. Yet, it is also possible that the rarity of such reports reflects a lack of data to test for genetic exchange, rather than an absence of such exchange. In discussing the dearth of examples of introgressive hybridization in entire clades of tropical birds, Grant and Grant [16] argued that the lack of examples from these groups might be due to cryptic morphological differences between species. Likewise, it is possible that in the marine realm reticulate evolution occurs at a similar frequency to that encountered for many non-marine clades, but the difficulty in collecting/observing the organisms has limited its detection [17]. Thus, the title of this review reflects the question of whether or not the marine realm reflects a “Final Frontier” in terms of testing for the role of reticulate evolution in the origin and trajectory of organismic lineages and assemblages.

In contrast to the rarity of reticulate evolution (whether biologically-based or due to lack of sampling) inferred for many marine clades, some researchers have invoked a major role for genetic exchange in the diversification of some taxa (*e.g.,* corals; [18,19]). Furthermore, analyses of fossil records for marine organisms also support the contention that reticulate evolution has been a characteristic of certain assemblages across time as well as phylogenetic and geographic space [20,21]. Finally, if the evolutionary effect of reticulation is tested across the extreme taxonomic diversity present in this biome, including microorganisms, it is predicted that gene transfer would be seen as having a fundamentally important role in the evolution of marine environments. Thus, like many clades that reside in terrestrial and freshwater habitats, the evolution of marine organisms may also be better reflected by a web-of-life metaphor rather than the tree-of-life concept [6,7,17,22,23]. In this review, we cite marine examples of reticulate evolution in organisms as diverse as archaebacteria, bacteria, seaweed, eelgrass, coral, shrimp, tuna and fur seals. These examples are not exhaustive. Rather, they are included to reflect the breadth of organisms that have evolved in the face of gene flow. Specifically, we present evidence of a significant effect from genetic exchange on the population genetic structure, evolutionary diversity, and adaptive evolution across all major groups of marine organisms (Table 1).

## 2. Horizontal Transfer

### 2.1. Horizontal Transfer and Adaptation in Marine Archaea, Bacteria and Cyanobacteria

Archaea and bacteria demonstrate evidence of extensive genetic exchange via horizontal gene transfers (Table 1). For example, DeLong [24] reported rDNA sequences characteristic of archaebacteria (*i.e.,* Archaea) in a previously unknown environment for these organisms, that of oxygenated coastal surface waters. This observation generated the following hypothesis: Eubacteria, Archaea and Eukarya “*…reside and compete in the ocean’s photic zone under the pervasive influence of light*” [25]. This hypothesis leads to several predictions, one of which is that if Archaea and Eubacteria are to compete for light-limited resources, they must both possess genes involved in the utilization of photic energy. It is therefore significant that such genes have been isolated from Archaebacteria and Eubacteria (*e.g.,* [25–27]). Specifically, photoproteins termed proteorhodopsins have been detected [26]. Furthermore, the detection of the shared genetic architecture for utilizing light energy has thus been ascribed to the lateral transfer of genes into the Archaea.

Frigaard *et al.* [25] also detected evidence for lateral transfer between planktonic bacteria and Archaea as well. This transfer was suggested to be adaptive in nature given that the proteorhodopsin genes isolated from euryarchaeotes were present in those isolates taken from photic, but not subphotic, regions of the water column. This finding was consistent with an adaptive scenario in which the organisms in the light-limited zones gained no benefit from the transfer of the photo-response genes, while those organisms living in the photic zone did [25]. From their findings, Frigaard *et al.* [25] made the general conclusion that “*…lateral gene dispersal mechanisms, coupled with strong selection for proteorhodopsin in the light, have contributed to the distribution of these photoproteins among various [marine] members of all three of life’s domains*.”

In addition to the above examples, cyanobacteria have also been shown to possess genetic components most likely resulting from horizontal transfer events. Shi and Falkowski [28]–in an examination of 682 loci–discovered widespread disagreement between phylogenies constructed for 13 cyanobacteria genomes. This discordance among phylogenetic hypotheses was apparently due to large-scale horizontal gene transfer. Indeed, of the 682 orthologs analyzed only 323 were placed into a “core set” whose evolutionary histories seemed congruent [28]. The majority of the loci thus appeared to have been potentially affected by genetic exchange. For example, it was hypothesized that the unique presence of the genetic architecture for nitrogen fixation originated from exchange with a heterotrophic prokaryotic lineage [28]. Likewise, Swingley *et al.* [29] also discovered evidence for the transfer of the genes (*i.e.,* to produce chlorophyll *d*) that provided the cyanobacterial species, *Acaryochloris marina*, with the ability to utilize far-red light for photosynthesis. The close physical association of the cyanobacterium with other oxygenic phototrophs (*e.g., Prochloron*) and the selective benefit (to the recipients) of being outside the competitive milieu of organisms possessing chlorophyll *a* and/or *b* likely facilitated the acquiring of this function [29].

### 2.2. Horizontal Transfer and the Evolution of the Marine Protist, Micromonas

Worden *et al.* [30] suggested that members of the picoeukaryotic genus, *Micromonas*, could play a role as “sentinel organisms” for monitoring climate-change driven perturbations in oceanic systems. This potential utility as biogeochemical-indicator species is due to their distribution, and thus adaptation, across tropical to polar marine ecosystems.

Significantly, the differentiation and adaptive evolution of various isolates belonging to the *Micromonas* clade is likely the result of the combined action of horizontally-acquired genes and selection. In particular, a large fraction of the genes identified as “unique” to these eukaryotes lineages analyzed shared significant similarity with clades of prokaryotes. Furthermore, the two isolates examined by Worden *et al.* [30] were highly divergent at these loci reflecting this combination of reticulation and differential selection. Indeed, this pattern of divergence and acquisition (via horizontal transfer) reflects the evolutionarily dynamic nature of these protist lineages [30], and also indicates the potential for genetic exchange to underlie adaptive evolution [5–7].

### 2.3. Horizontal Transfer of Transposable Elements among Marine Invertebrates

The exchange of transposable elements (*i.e.,* transposons) among distantly-related terrestrial organisms has been recognized for more than a decade (reviewed in [31]). For example, the relatively recent introduction of transposons known as “Type II class elements” was detected for the cosmopolitan invertebrate, *Drosophila melanogaster*. Likewise, a recent horizontal exchange of this class of element was inferred between *D. melanogaster* and *D. willistoni*, two species that last shared a common ancestor >50 million years ago [31].

Unlike the terrestrial lineages mentioned above, genomic information for marine invertebrates is relatively limited [32]. Tests for the horizontal exchange of transposable elements have been limited by this lack of genomic data. However, recent work has not only identified various Type II transposons, but has also identified instances of apparent horizontal exchange between phylogenetically-unrelated organisms that occur in close spatial proximity. Specifically, Casse *et al.* [32] identified *mariner*-like elements in the genomes of four different marine invertebrates (Table 1). Two of the species, *Cancer pagurus* and *Maia brachydactila*, are coastal crustaceans, while the remaining two species, *Ventiella sulfuris* and *Bythograea thermydron* are hydrothermal vent-associated organisms (an amphipod and crab, respectively [32]).

Though phylogenetically highly divergent lineages, the *mariner*-like elements isolated from the genomes of the coastal and the hydrothermal vent species demonstrated high levels of sequence similarity to the organism with which it was spatially associated [32]. In particular, the two hydrothermal organisms possessed elements that shared 99.5% similarity. Transposable elements isolated from the two coastal species likewise exhibited >99% sequence similarity. These findings led to the conclusion that, as for terrestrial invertebrates, horizontal transfer had resulted in the exchange of the Type II class elements between the unrelated, but sympatrically-distributed, coastal and hydrothermal vent species [32].

## 3. Introgressive Hybridization and Hybrid Speciation

### 3.1. Introgressive Hybridization in Marine Angiosperms

Table 1 lists three examples of marine plant clades that demonstrate the effect of introgressive hybridization and hybrid speciation (both homoploid–*i.e.,* diploid hybrid derivative lineages–and polyploid). Of these, we will consider only the eelgrass, *Zostera*. This aquatic angiosperm, like other seagrasses, is an important constituent of the marine coastal communities in which it occurs. Though eelgrass genotypes have the capacity to reproduce asexually via rhizomatous growth, they also reproduce sexually, with their seeds having the ability to be transported over long distances by rafting on detached plants (*e.g.,* up to 50km along the Northern European coast [33]).

Pollen flow among eelgrass populations appears limited (reviewed in [33]); however, gene flow via male gametes does occur at low frequencies. For example, in a sample of 28 *Zostera* “meadows” located both in the California Channel Islands and the adjacent mainland, Coyer *et al.* [34] detected genetic variation indicative of significant levels of gene flow, particularly for the coastal populations. Furthermore, their samples from the eelgrass sites included two species identified as *Zostera marina* and *Z. pacifica*. In addition to the genetic connectivity caused by clonal growth and sexual reproduction, these samples reflected admixtures of the two species’ genomes. Specifically, the assignment of genotypes to classes of *Z. marina, Z. pacifica*, or “introgressed”, utilizing microsatellite marker loci, detected hybrid/parental assemblages at the Santa Catalina, San Clemente and San Diego sites [34]. Each of these sites was suggested as a possible example of anthropogenically-mediated introgressive hybridization thus reflecting the extensive impact of humans on coastal environments. These results also led to the conclusion that introgressive hybridization may occur throughout the global distribution of *Zostera* populations, thereby contributing to the genetic variability in numerous eelgrass species [34].

### 3.2. Introgressive Hybridization and Hybrid Speciation in Corals

The literature concerning the role of reticulate evolution in the origin and diversification of some coral clades is extensive. For example, descriptions of widespread introgressive hybridization leading to an enrichment of genetic and morphological variation are replete for reef corals belonging to the genus *Acropora* [19]. In this regard, Hatta *et al.* [35] found high rates of experimental, interspecific fertilization between naturally hybridizing species of acroporids. In contrast, Knowlton *et al.* [36] and Levitan *et al.* [37] defined barriers to reproduction between different species and morphotypes of *Montastraea* corals, but with regional differences in the strength of isolation. In addition, Fukami *et al.* [38] detected a north to south hybridization gradient in these same *Montastraea* lineages using molecular and morphological analyses. This latter study provided evidence that introgression between these species occurred mostly in the northern portion of their distribution. Likewise, an analysis of the family Faviidae documented extensive paraphyly across numerous clades [39]. It was suggested that introgressive hybridization may have contributed to the observation of paraphyly. Specifically, Huang *et al.* [39] argued “*Introgression…may have resulted in such disparity, where the gene tree does not resemble the species tree, and neither is well-correlated with morphological evolution…*” Additional evidence for introgressive hybridization within this clade of corals has also come from the fossil record. Specifically, morphological variation across the fossil record of this coral genus led to the inference of introgressive hybridization during the Pleistocene period [21].

Examples of hybrid speciation and introgression affecting coral evolution have been found within the genera *Alcyonium* and *Pocillopora* as well. McFadden & Hutchinson [40] tested the hypothesis of hybrid speciation giving rise to members of two genera of European soft corals (*i.e., Alcyonium* and *Bellonella*). Specifically, they analyzed sequence variation in the rRNA internal transcribed spacer (“*ITS-1*”) region of the putative hybrid lineages, *Alcyonium hibernicum* and *Bellonella bocagei*. The following patterns of genetic variability did indeed support the hypothesis of hybrid derivation for each of these lineages: (1) *A. hibernicum* possessed a combination of two different sequence variants that were characteristic of divergent clades of soft corals, and (2) *B. bocagei* possessed divergent *ITS-1* types inferred to be recombinants between the same two divergent sequence families [40].

Species belonging to the tropical eastern Pacific scleractinian coral genus, *Pocillopora*, are the dominant reef-building organisms in this region [41]. Unlike their congeners found throughout most of the genus’ geographical distribution, these species have shifted from internal brooding of larvae to free-spawning [42]. Interestingly, the transition in reproductive mode was correlated with introgressive hybridization among five species of these tropical corals. Like the study of *Alcyonium* and *Bellonella*, rRNA *ITS* sequence data were collected from *Pocillopora* individuals distributed in the tropical eastern Pacific [41]. These data revealed a sharing of sequence variants among *Pocillopora damicornis*, *P. eydouxi*, *P. elegans*, *P. inflata* and *P. effusus*. Not only did Combosch *et al.* [41] infer introgressive hybridization as the source of this genetic variation, but they also concluded that the gene flow was largely unidirectional, with *P. damicornis* acting as the recipient of the allelic variability. Thus, numerous coral assemblages, including Indo-Pacific acroporids, the European soft corals and the eastern Pacific *Pocillopora* clades, reflect signatures of reticulate evolution.

### 3.3. Introgressive Hybridization in Protists–The Diatom Genus Pseudo-nitzschia

Diatoms of the genus *Pseudo-nitzschia* are probably best known as the organisms responsible for ‘harmful algal blooms’ (HABs) during which large quantities of the neurotoxin, domoic acid is produced [43]. Domoic acid is the causative agent of ‘amnesic shellfish poisoning’ (ASP), a syndrome often caused (as the name suggests) by ingestion of shellfish laden with this neurotoxin [43]. Such poisoning events prior to the 1980’s had been ascribed mainly to toxin-producing dinoflagellate or cyanobacteria, taken up by shellfish that were subsequently eaten by humans [43]. However, a particular episode of poisoning in 1987 led to the discovery of *Pseudo-nitzschia* as the source of a low molecular weight amino acid (*i.e.,* domoic acid) leading to ASP [43].

At least 12 species of *Pseudo-nitzschia* are now known to have the capacity to produce domoic acid (reviewed in [43,44]). Most of these species are now believed to be cosmopolitan [43,44], with genetic and morphological data indicative of multiple strains and/or varieties within some of the species [45,46]. For the present discussion it is significant that the evolution of multiple strains/varieties and their occurrence in sympatric associations have led to introgressive hybridization. This introgression has been documented using a variety of molecular and morphological markers. For example, D’Alelio *et al.* [45] detected admixtures of the *Pseudo-nitzschia multistrata* strains “A” and “B” (using the internal transcribed spacer region (ITS) of the ribosomal RNA genes) in samples from the Gulf of Naples. They thus found individual genomes defined as having A + B haplotypes. Because the individuals belonging to the A and B categories could not be defined on the basis of any other molecular or morphological parameter examined, it was concluded that this pattern of ITS admixture was due to the overlap of conspecific, but somewhat divergent, populations that had arisen either *in situ* or allopatrically [45].

Like the results from *P. multistrata*, analyses of *P. pungens* populations also detected patterns indicative of both divergence and introgression [46]. However, unlike *P. multistrata*, the *P. pungens* lineages were characterized by divergence in multiple genomic (*i.e.,* ITS and chloroplast loci) and morphological characters [46]. Furthermore, the degree of divergence in the DNA and morphological characters allowed a relatively detailed definition of introgressive hybridization in a hybrid zone in the northeast Pacific. Casteleyn *et al.* [46] detected individuals exhibiting mixtures of morphological traits and chloroplast/nuclear haplotypes indicative of both first-generation and advanced-generation hybrids. As with all of the other examples given in this review, the detection of introgression within *P. multistrata* and *P. pungens*, suggests a broader base of genetic exchange among marine organisms than has been previously appreciated.

### 3.4. Introgressive Hybridization in Crustacea

#### 3.4.1. Genus Tetraclita (Acorn Barnacles)

The predominant intertidal barnacle lineages in the northwestern Pacific Ocean belong to the genus *Tetraclita*. Because two of these “acorn barnacles” have been found to possess identical mitochondrial haplotypes, as well as very similar morphologies, they were recently reduced from specific to subspecific status. Tsang *et al.* [47] tested the genetic distinctiveness and the geographic pattern of genetic variation of these two subspecies (*Tetraclita japonica japonica* and *T. j. formosana*) using amplified fragment length polymorphisms. Tsang *et al.*’s [47] analysis led to the following series of observations and hypotheses: (1) warming in the oceans may have been the catalyst for poleward movement of some of the acorn barnacle lineages belonging to the genus *Tetraclita*; (2) *T. j. formosana* migrated to Japan and successfully colonized habitats there; (3) following this migration to Japan, and because of their relative scarcity, the *T. j. formosana* individuals mated frequently with the more numerous *T. j. japonica* barnacles; (4) the bouts of hybridization in Japan have resulted in numerous F_1_ and backcross hybrid individuals; (5) likewise, introgressive hybridization has also occurred in Okinawa; and (6) continued migration fueled by shifts in oceanic temperatures may lead to the genetic assimilation and thus disappearance of some of the *Tetraclita* lineages. Thus, although *T. j. japonica* and *T. j. formosana* were confirmed as genetically-differentiated lineages worthy of recognition, this distinctiveness may be lost if migration causes greater genetic admixture between these taxa [47].

#### 3.4.2. Genus Mysis (Opossum Shrimp)

Opossum shrimp species belonging to the genus *Mysis* are distributed throughout aquatic habitats. Because they are found throughout the world’s marine and freshwater zones they have been used as a model system for testing hypotheses concerning the origin and evolutionary trajectories of geographically disjunct, but phylogenetically-related, zoogeographic elements [48]. Though questions concerning the processes that have affected current day distributions of such disjunct clades remain (*e.g.,* [49]), studies of the opossum shrimp have defined phylogenetic signatures suggesting the role of reticulation in their evolutionary history.

Audzijonyte *et al.* [48] reconstructed the phylogenetic relationships among ca. 15 species of *Mysis* that possessed either circumarctic, northwest Atlantic, Continental or Caspian Sea distributions. The data that were used for the phylogenetic reconstructions included morphological characters and nuclear/mtDNA sequences. Instances suggestive of mtDNA introgression following divergence included (1) the Caspian Sea assemblage, (2) the circumarctic species, *Mysis litoralis* and *M. oculata* and (3) the continental species, *M. salemaai* and *M. segerstralei* [48]. In each case, the evidence for reticulate evolution came from phylogenetic discordance between evolutionary trees derived from different data sets.

A more recent analysis of morphological and genetic variation among *Mysis* species also resulted in the inference of post-divergence introgression. In this latter study, Audzijonyte and Väinölä [50] examined the divergence among the three circumpolar species, *M. nordenskioldi, M. litoralis* and *M. oculata*. Though previously difficult to separate, these three species were found to possess diagnostic combinations of morphological and genetic characteristics. However, Audzijonyte *et al.* [48] and Audzijonyte and Väinölä [50] also detected discordances. In particular, the three species were distinguishable using both morphology and nuclear loci, but *M. litoralis* and *M. oculata* formed an unresolved cluster based upon mtDNA variability. Once again, these data support the hypothesis of post-divergence, mtDNA introgression among *Mysis* lineages [50].

### 3.5. Introgressive Hybridization between Hydrothermal Vent Mussels of the Genus, Bathymodiolus

Like marine groups such as corals and cyanobacteria, mussels have a rich literature indicating extensive genetic exchange between divergent lineages (Table 1). For example, Arnold [5,7] has reviewed in detail work documenting the effect of introgression on the genetic structure of widely dispersed species of the genus, *Mytilus*. However, reticulate evolution is not limited to these near-shore taxa.

O’Mullan *et al.* [51] and Won *et al.* [52] reported genetic analyses of deep-sea hydrothermal vent mussels across an area of sympatry between the species, *Bathymodiolus azoricus* and *B. puteoserpentis*. Both studies detected introgressed individuals along a ridge in the area of overlap between the northern *B. azoricus* and southern *B. puteoserpentis*. In the first of the analyses, morphometric and genetic data (from both nuclear and mtDNA loci) “*revealed a mixed population with gene frequencies and morphology that were broadly intermediate to those of the northern and southern species…*” [51]. The spatially restricted nature of the hybrid individuals suggested the presence of selection against at least some of the hybrid genotypes. This latter hypothesis was supported by cytonuclear disequilibrium estimates [52]. In particular, Won *et al.* [52] discovered a pattern indicative of parental migration into the zone and restriction of the hybrids to the zone of overlap due to selectively disadvantageous interactions between genes inherited from the two species [52]. Notwithstanding the evidence for selection acting against some hybrid genotypes, recombination between the two hydrothermal vent mussel genomes–at least within the hybrid zone–was apparent.

### 3.6. Introgressive Hybridization in Echinodermata

#### 3.6.1. Sea Urchin Species

Numerous evolutionary studies involving various clades of sea urchins have defined reproductive barriers between congeners. For example, Levitan [53] reported the degree to which eggs from three species of *Strongylocentrotus* (*i.e., droebachiensis, franciscanus, purpuratus*) could be fertilized with either conspecific or heterospecific sperm. Levitan [53] found that eggs from females most easily fertilized with conspecific sperm (*e.g., S. droebachiensis*) were also less discriminating towards sperm from other species. Thus, Levitan [53] found a cline of reproductive isolation from *S. droebachiensis* (least isolated) to *S. franciscanus* (intermediate isolation) to *S. purpuratus* (highly isolated). Furthermore, Harper *et al.* [54], in an analysis of gene flow within and between species of *Strongylocentrotus* sea urchins, concluded that sympatric populations of different species exchanged genes at much lower frequencies than did populations of the same species separated by oceans.

In spite of reproductive barriers between sea urchin lineages, introgressive hybridization has been well documented between numerous species and subspecies (Table 1). Indeed, even the clades utilized to define reproductive isolation (*e.g., Strongylocentrotus*) contain genetic variation consistent with reticulate evolution. For example, both Addison and Hart [55] and Harper *et al.* [54] described extensive interspecific gene flow between *S. droebachiensis* and *S. pallidus* throughout the range of the former species (in the northwest Atlantic and Pacific oceans). Likewise, Lessios and Pearse [56] produced evidence of echinoid introgressive hybridization from a combined genetic and morphological analysis of *Diadema paucispinum, D. savignyi* and *D. setosum*. Specifically, these authors detected genotypes that suggested introgression between combinations of all three of the *Diadema* lineages. Finally, Zigler and Lessios [57] also reported variability at mitochondrial and nuclear loci demonstrative of introgression within the genus *Lytechinus*; intersubspecific and interspecific introgression was detected between *Lytechinus variegatus variegatus*/*L. v. carolinus* and *L. variegatus*/*L. williamsi*, respectively [57].

#### 3.6.2. Genus Asterias (Sea Stars)

As with sea urchins of the genus *Strongylocentrotus*, Harper *et al.* [54] also detected patterns of genetic variation in sea stars reflective of long-distance gene flow within species, but a more limited effect from introgression between sympatric species. Yet, introgression does indeed affect the genetic structuring of *Asterias* species that co-occur and thus form hybrid zones. For example, both Harper & Hart [58] and Scheibling and Lauzon-Guay [59] reported morphological and/or mtDNA data demonstrating contemporary hybrid zones between *Asterias forbesi*/*A. rubens* and *A. vulgaris*/*A. forbesi*/*A. rubens*, respectively. Both of these above studies analyzed a zone of overlap in the northwest Atlantic. In the analysis by Harper and Hart [58], mtDNA and morphological data were collected and the resulting patterns of phenotypic and mtDNA variation were compared for several populations. The morphological characters suggested only two groups, reflective of *A. forbesi* and *A. rubens* phenotypes. However, the mtDNA sequence variability collected from the same populations was discordant with the morphology and suggested the presence of advanced-generation hybrids not detectable with quantitative (*i.e.,* morphological) characters [58]. In contrast, a study based upon morphological characters diagnostic for *A. vulgaris*, *A. forbesi* and *A. rubens* did detect variation indicating a mosaic of “*A. rubens*” and “*A. forbesi*” phenotypes [59]. Furthermore, some individuals possessed morphological traits suggesting introgression involving *A. vulgaris* as well [59].

### 3.7. Introgressive Hybridization and Hybrid Speciation in Coral Reef Fishes Belonging to the Genera, Plectropomus and Acanthurus

Table 1 reflects the growing literature indicating the role of reticulate evolution within several different species complexes commonly known as coral reef fish. Two of these clades–*Plectropomus* and *Acanthurus*–exemplify the outcomes of introgressive hybridization and introgressive hybridization/hybrid speciation, respectively [60,61]. In particular, member lineages of these unrelated genera possess mosaic genomes and/or phenotypes reflecting contributions from multiple species. Furthermore, some of these hybrid lineages have been recognized as species.

van Herwerden *et al.* [60] defined the nuclear and mtDNA variation in two species of grouper, *Plectropomus maculatus* and *P. leopardus*, found along both the eastern and western Australian coastlines. The patterns of phylogenetic differentiation led these workers to infer both introgressive hybridization and incomplete lineage sorting as causal for discordances among the genetic markers. In particular, (1) the lack of reciprocal monophyly in mtDNA phylogenies for the eastern populations of the two species, but (2) the resolution of species-specific clades for the western samples, suggested an impact of introgression on the east coast lineages [60]. In contrast, incomplete lineage sorting was inferred as the cause of the discordances found among the nuclear-based phylogenies. Thus, one of three loci generated clades containing only one of the species. Two of the nuclear loci produced admixed clades containing samples from both *P. maculatus* and *P. leopardus* [60]. It is, however, possible that the discordance among the nuclear phylogenies, like those from the mtDNA, could also reflect the role of introgression.

**Figure 2.**
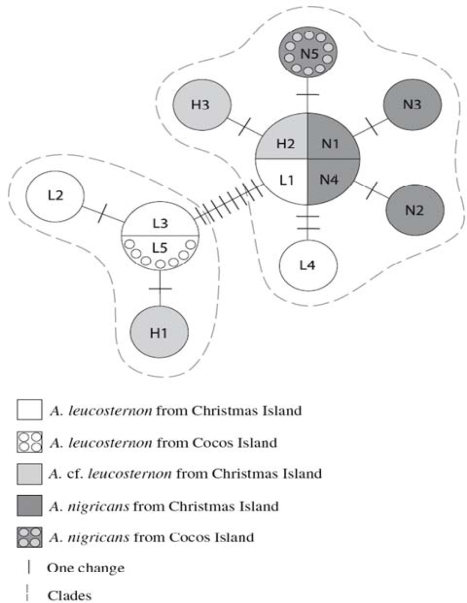
Genetic associations (based upon mtDNA sequence variation) between surgeonfish categorized as hybrids (“*Acanthurus* cf. *leucosternon*”), *A. leucosternon* or *A. nigricans.* The relative sizes of the circles reflect the number of individuals sharing a particular mtDNA haplotype. Bars on the lines connecting haplotypes indicate the number of substitutions differentiating them. Dashed lines surround the two major *Acanthurus* clades [61]. Reprinted with permission from Springer [61].

A study of genetic variation in an area of sympatry in the eastern Indian Ocean between the coral reef surgeonfish, *Acanthurus leucosternon* and *A. nigricans*, also resolved patterns indicative of reticulate evolution (Figure 2). Marie *et al.* [61] collected DNA sequence data for three distinctive morphotypes, two reflecting *A. leucosternon* and *A. nigricans* and the third being a hypothesized hybrid species (*i.e.,* “*A.* cf. *leucosternon*”). Sequence information was obtained from both mtDNA and nuclear loci. These data allowed simultaneous tests for introgression between *A. leucosternon* and *A. nigricans*, and the hybrid origin of *A.* cf. *leucosternon*. Both the introgression and hybrid speciation hypotheses were supported by the mtDNA data [61]. First, admixed clades of all three species were defined by the mtDNA sequence information (Figure 2). Indeed, the extent and directionality of introgression suggested concern that the *A. leucosternon* lineage might be lost from the region of overlap [61]. Second, mtDNA haplotypes characteristic of allopatric populations of both *A. leucosternon* and *A. nigricans* were detected in the sample of *A.* cf. *leucosternon* individuals (Figure 2); this is consistent with a hybrid origin for the “intermediate color patterns” possessed by A. cf. leucosternon [61].

### 3.8. Introgressive Hybridization in Marine Turtles

Reticulate evolution in the form of introgressive hybridization has been well defined for numerous marine turtle taxa. For example, Karl *et al.* [62] used an analysis of both mtDNA and nuclear loci to test for the infrequent formation of hybrids among loggerhead, Kemp’s ridley, hawksbill and green sea turtles (*Caretta caretta, Lepidochelys kempii, Eretmochelys imbricata* and *Chelonia mydas*, respectively). Likewise, Bass *et al.* [63] detected divergent mtDNA haplotypes in Brazilian samples of hawksbill turtles that were identical, or nearly identical, to those found in loggerhead samples. Each of these analyses thus suggested the likelihood of low-frequency introgression in a number of marine turtle clades. Furthermore, the findings of Bass *et al.* [63] indicated the possibility of a large effect from introgressive hybridization on some lineages; 10 of 14 Brazilian “hawksbill” animals possessed mtDNA haplotypes most similar to loggerhead turtles.

Recently, Lara-Ruiz *et al.* [64] analyzed hawksbill populations from the state of Bahia in Brazil. This region contains > 90% of the *E. imbricata* nesting sites in Brazil. The high frequency of introgression in the Brazilian hawksbill samples suggested a decade earlier by Bass *et al.* [63] was confirmed by the much larger sample of 119 individuals. Over half of the turtles sampled (*i.e.,* 67) possessed the expected mtDNA sequences characteristic of *E. imbricata*. Yet, of the remaining 52 individuals, 50 reflected introgression of mtDNA from *C. caretta* (loggerheads), while two possessed mtDNA from *L. olivacea* (*i.e.,* the olive ridley lineage; [64]). Thus, introgressive hybridization among marine turtle lineages is taxonomically diverse and, in some cases, extensive in terms of the proportion of the population impacted.

### 3.9. Introgressive Hybridization in Fur Seals

Human-mediated environmental changes have been demonstrated to be catalysts for bouts of genetic exchange among a diverse array of organisms (see [6,7] for reviews). In this regard, introgressive hybridization among species of the fur seal genus, *Arctocephalus* reflects the role of anthropogenic processes [65,66]. Analyses of various fur seal populations thus suggested that the observed introgression was at least partly caused by the extinction (or near-extinction) of populations of *Arctocephalus gazella, A. tropicalis* and *A. forsteri* (Antarctic, subantarctic and New Zealand fur seals, respectively) due to human harvesting. The introgression among the fur seals was hypothesized to have occurred due to the recolonization of islands by multiple species occupied formerly by a single taxon [65,66].

**Figure 3.**
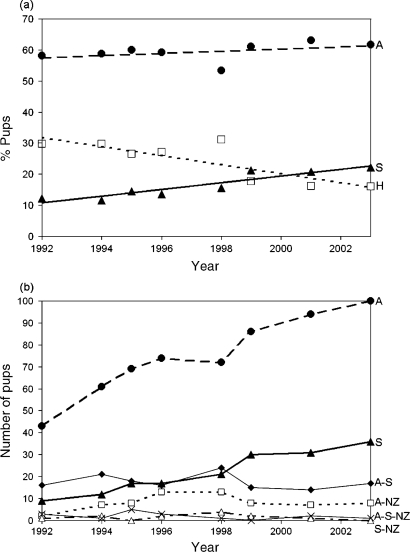
Change over time in a) the percentage of Antarctic (“A”), subantarctic (“S”) and hybrid (“H”) fur seal pups and b) the numbers of A, S and H pups, including values for the four hybrid classes (*i.e.,* Antarctic x subantarctic, “A-S”; Antarctic x New Zealand, “A-NZ”; subantarctic x New Zealand, “S-NZ”; Antarctic x subantarctic x New Zealand, “A-S-NZ”; from [65]). Reproduced with permission from Wiley-Blackwell [65].

In their analysis of the genetic structure of Macquarie Island, Lancaster *et al.* [65] collected mtDNA and nuclear sequence variation for 1007 pups sampled over an eight year period (Figure 3). Though *A. gazella* genotypes predominated, hybrids among all three species were also detected, with the percentage of hybrid pups averaging ca. 23% and varying from 17–30% across years (Figure 3). Four hybrid categories were identifiable from the Macquarie Island samples. These included: (1) *A. gazella* x *A. tropicalis*; (2) *A. gazella* x *A. forsteri*, (3) *A. tropicalis* x *A. forsteri* and (4) *A. gazella* x *A. tropicalis* x *A. forsteri* (Figure 3) [65]. Though lower reproductive success was detected for some of the hybrid classes, the presence of “multiple mating strategies” have led to the establishment of this genetically diverse hybrid zone among the three fur seal taxa [67,68]. Likewise, Kingston and Gwilliam [66] also detected introgression on Iles Crozet, but only between subantarctic and Antarctic fur seals. Furthermore, their estimated frequencies of hybridization were lower than that of Lancaster *et al.* [65]; these authors estimated the frequency of F_1_ hybrids at 1% of the total population and 1.6% of the pups. They also concluded that at least 2.4% (and possibly as much as 4%) of the population consisted of backcross progeny [66].

## 4. Conclusions and Future Directions

We believe that the above examples (and those given in previous reviews, see [17]) indicate that reticulate evolution (as reflected by introgressive hybridization, hybrid speciation and horizontal transfer events) is not limited to a few categories of marine organisms. Indeed, though reproductive isolation is a key factor in the speciation process–reducing genetic exchange in areas of geographic overlap between closely related organisms–divergence of marine lineages in the face of gene flow (*i.e.,* sympatric or parapatric divergence) is indicated for a wide array of organisms (Table 1). Thus, genetic exchange and evolutionary diversification appear to reflect simultaneous processes during many radiations of marine clades. Furthermore, not only has genetic exchange occurred, but also this exchange has been associated with the origin of new lineages and, in some cases, the transfer of adaptations leading to the invasion of new habitats (*e.g.,* Archaebacteria).

All of the above observations belie the conclusion that genetic exchange involving marine assemblages is relatively rare compared to that observed for terrestrial organisms. In addition, the origins of novel lineages and/or adaptations via hybridization and horizontal transfer indicate the potential evolutionary significance of exchange between divergent marine lineages. Yet, questions remain. Most importantly it remains to be seen whether there is a relationship between the clade to which an organism belongs and the potential for genetic exchange? The relevant data suggest that the genomic architecture, adaptive potential and thus ecological and evolutionary diversification of prokaryotic organisms in the marine realm, like their terrestrial counterparts, have been greatly affected by gene acquisition via horizontal gene transfer. Furthermore, some might argue that plant clades would also demonstrate a high proportion of reticulate events. But, what of animal lineages? As already mentioned, some authors (including MLA) have previously argued against a prominent role for introgressive hybridization among marine animals. Indeed, there are still few data sets to test for genetic exchange among such groups–though those that exist uniformly detect patterns that reject the strictly allopatric model of divergence and the application of the biological species concept as a robust descriptor of evolutionary pattern and process. Thus, as with most evolutionary hypotheses, those that predict the frequency, phylogenetic distribution and effect of genetic exchange on adaptive change and speciation in marine organisms necessitate additional, detailed studies of the genetic/genomic constitution of diverse lineages of prokaryotes and eukaryotes.

## Figures and Tables

**Table 1.** Selected examples of marine organisms for which genetic exchange events (*i.e.,* horizontal transfer, introgressive hybridization and/or hybrid speciation) have been inferred. Lineages that are either the donor or recipient of DNA sequences are included. The genus, species and common name (if available) for each example are given. In addition, whether the genetic exchange was characterized as horizontal transfer (as, for example, is the case in transfers between cyanobacteria and bacteriophages) or introgressive hybridization (*i.e.,* involving sexual reproduction and backcrossing; 1) is noted. The term “Multiple” is given under the “Taxon” category to indicate the interaction of more than one lineage from the taxonomic group. For example, the categories of “Cyanobacteria” and “Bacteriophage” reflect analyses of at least 14 species/strains and ca. 100 divergent viral lineages, respectively. The type of analyses used (morphological analyses, population genetic surveys, genome sequence analyses and/or tests for phylogenetic discordance) to infer the exchange events are also indicated, along with the reference(s) that reported the findings. Note: the final example listed comes from fossil data and combines results from studies of crinoids and corals.

**Taxon**	**Common Name**	**Type of Genetic Exchange**	**Data**	**Reference(s)**
Multiple	Cyanobacteria	Horizontal transfer	Genome sequence analyses, Phylogenetic discordance	[28,29,69–73]
Multiple	Bacteriophage	Horizontal transfer	Genome sequence analyses, Phylogenetic discordance	[72–74]
Multiple	Bacteria	Horizontal transfer	Genome sequence analyses, Phylogenetic discordance	[25]
Multiple	Archaebacteria	Horizontal transfer	Genome sequence analyses, Phylogenetic discordance	[25]
*Spartina* (Multiple)	Cordgrass	Introgressive hybridization, Hybrid speciation	Population genetic surveys, Phylogenetic discordance	[75–79]
*Zostera* (Multiple)	Eelgrass	Introgressive hybridization	Population genetic surveys	[34]
*Fucus* (Multiple)	Seaweed	Introgressive hybridization, Hybrid speciation	Population genetic surveys, Phylogenetic discordance	[80–84]
*Pseudo-nitzschia pungens*	Diatom	Introgressive hybridization	Morphological analyses, Population genetic surveys	[44–46]
*Bythograea thermydron*	Hydrothermal crab	Horizontal transfer (transposable elements)	Phylogenetic discordance	[32]
*Ventiella sulfuris*	Hydrothermal amphipod	Horizontal transfer (transposable elements)	Phylogenetic discordance	[32]
*Maia brachydactila*	Sea shoe	Horizontal transfer (transposable elements)	Phylogenetic discordance	[32]
*Cancer pagurus*	Crab	Horizontal transfer (transposable elements)	Phylogenetic discordance	[32]
*Menippe* (Multiple)	Stone crab	Introgressive hybridization	Population genetic surveys	[85–87]
*Mysis* (Multiple)	Opossum shrimp	Introgressive hybridization	Phylogenetic discordance	[48,50]
*Diadema* (Multiple)	Sea urchins	Introgressive hybridization	Population genetic surveys	[56]
*Lytechinus* (Multiple)	Sea urchins	Introgressive hybridization	Population genetic surveys, Phylogenetic discordance	[57]
*Strongylocentrotus* (Multiple)	Sea urchins	Introgressive hybridization	Population genetic surveys, Phylogenetic discordance	[53–55]
*Asterias* (Multiple)	Sea stars	Introgressive hybridization	Morphological analyses, Population genetic surveys, Phylogenetic discordance	[54,58,59]
*Acrocnida brachiata*	Brittle-star	Introgressive hybridization	Population genetic surveys	[88]
*Alcyonium hibernicum*	Coral	Hybrid speciation	Population genetic surveys, Phylogenetic discordance	[40]
*Bellonella bocagei*	Coral	Hybrid speciation	Population genetic surveys, Phylogenetic discordance	[40]
*Pocillopora* (Multiple)	Corals	Introgressive hybridization	Population genetic surveys, Phylogenetic discordance	[41]
*Acropora* (Multiple)	Corals	Introgressive hybridization, Hybrid speciation	Morphological analyses, Population genetic surveys, Phylogenetic discordance	[19,35,89–94]
*Littorina saxtilis*	Snail	Introgressive hybridization	Morphological analyses	[95,96]
*Mercenaria* (Multiple)	Clams	Introgressive hybridization	Population genetic surveys	[97,98]
*Macoma balthica*	Clam	Introgressive hybridization	Population genetic surveys	[99,100]
*Crassostrea virginica*	American oyster	Introgressive hybridization	Population genetic surveys	[101,102]
*Mytilus* (Multiple)	Mussels	Introgressive hybridization	Morphological analyses, Population genetic surveys, Phylogenetic discordance	[103–114]
*Bathymodiolus* (Multiple)	Hydrothermal vent mussels	Introgressive hybridization	Morphological analyses, Population genetic surveys	[51,52]
*Scophthalmus maximus*	Turbot	Introgressive hybridization	Population genetic surveys	[115]
*Clupea harengus*	Atlantic herring	Introgressive hybridization	Population genetic surveys	[116]
*Gadus morhua*	Atlantic cod	Introgressive hybridization	Population genetic surveys	[117,118]
*Platichthys flesus*	European flounder	Introgressive hybridization	Population genetic surveys	[119]
*Pleuronectes platessa*	Plaice	Introgressive hybridization	Population genetic surveys	[119]
*Thunnus* (Multiple)	Tuna and Albacore	Introgressive hybridization	Population genetic surveys, Phylogenetic discordance	[120–122]
*Anguilla* (Multiple)	Eels	Introgressive hybridization	Population genetic surveys	[123,124]
*Sebastosomus* (Multiple)	Rockfish	Introgressive hybridization	Population genetic surveys	[125]
*Acanthochromis* (Multiple)	Damselfish	Introgressive hybridization	Population genetic surveys	[126,127]
*Plectropomus* (Multiple)	Coral trout	Introgressive hybridization	Population genetic surveys, Phylogenetic discordance	[60]
*Acanthurus* (Multiple)	Surgeonfish	Introgressive hybridization, Hybrid speciation	Population genetic surveys, Phylogenetic discordance	[61]
*Chaetodon* (Multiple)	Butterflyfish	Introgressive hybridization	Population genetic surveys, Phylogenetic discordance	[128]
*Sebastes* (Multiple)	Redfish	Introgressive hybridization	Population genetic surveys	[15]
Salmonidae (Multiple)	Charr, Salmon	Introgressive hybridization	Population genetic surveys, Phylogenetic discordance	[129,130]
*Caretta caretta*	Loggerhead turtle	Introgressive hybridization	Population genetic surveys, Phylogenetic discordance	[62–64]
*Lepidochelys* (Multiple)	Kemp’s ridley and Olive ridley turtles	Introgressive hybridization	Population genetic surveys, Phylogenetic discordance	[62–64]
*Eretmochelys imbricata*	Hawksbill turtle	Introgressive hybridization	Population genetic surveys, Phylogenetic discordance	[62–64]
*Chelonia mydas*	Green turtle	Introgressive hybridization	Population genetic surveys	[62]
*Arctocephalus* (Multiple)	Fur seals	Introgressive hybridization	Morphological analyses, Population genetic surveys	[65–68]
*Eretmocrinus* (Fossils)	Crinoids	Introgressive hybridization	Morphological analyses	[20]
*Montastraea* (Fossils)	Corals	Introgressive hybridization	Morphological analyses	[21]
